# Breeding farm, level of feeding and presence of antibiotics in the feed influence rabbit cecal microbiota

**DOI:** 10.1186/s42523-020-00059-z

**Published:** 2020-11-02

**Authors:** María Velasco-Galilea, Miriam Guivernau, Miriam Piles, Marc Viñas, Oriol Rafel, Armand Sánchez, Yuliaxis Ramayo-Caldas, Olga González-Rodríguez, Juan P. Sánchez

**Affiliations:** 1 Institute of Agrifood Research and Technology (IRTA) – Animal Breeding and Genetics, E08140 Caldes de Montbui, Barcelona, Spain; 2 Institute of Agrifood Research and Technology (IRTA) - Integral Management of Organic Waste, E08140 Caldes de Montbui, Barcelona, Spain; 3 Animal Genomics Group, Centre for Research in Agricultural Genomics (CRAG) CSIC-IRTA-UAB-UB, Campus UAB, Catalonia, Spain; 4 Unit of Animal Science, Department of Animal and Food Science, Autonomous University of Barcelona, Barcelona, Spain

**Keywords:** Cecal microbiota, Meat rabbit, Breeding farm, Feed restriction, Antibiotics, 16S MiSeq Illumina sequencing, Analysis of variance, Multivariate approach

## Abstract

**Background:**

The effect of the production environment and different management practices in rabbit cecal microbiota remains poorly understood. While previous studies have proved the impact of the age or the feed composition, research in the breeding farm and other animal management aspects, such as the presence of antibiotics in the feed or the level of feeding, is still needed. Characterization of microbial diversity and composition of growing rabbits raised under different conditions could help better understand the role these practices play in cecal microbial communities and how it may result in different animal performance.

**Results:**

Four hundred twenty-five meat rabbits raised in two different facilities, fed under two feeding regimes (ad libitum or restricted) with feed supplemented or free of antibiotics, were selected for this study. A 16S rRNA gene-based assessment through the MiSeq Illumina sequencing platform was performed on cecal samples collected from these individuals at slaughter. Different univariate and multivariate approaches were conducted to unravel the influence of the different factors on microbial alpha diversity and composition at phylum, genus and OTU taxonomic levels. The animals raised in the facility harboring the most stable environmental conditions had greater, and less variable, microbial richness and diversity. Bootstrap univariate analyses of variance and sparse partial least squares-discriminant analyses endorsed that farm conditions exerted an important influence on rabbit microbiota since the relative abundances of many taxa were found differentially represented between both facilities at all taxonomic levels characterized. Furthermore, only five OTUs were needed to achieve a perfect classification of samples according to the facility where animals were raised. The level of feeding and the presence of antibiotics did not modify the global alpha diversity but had an impact on some bacteria relative abundances, albeit in a small number of taxa compared with farm, which is consistent with the lower sample classification power according to these factors achieved using microbial information.

**Conclusions:**

This study reveals that factors associated with the farm effect and other management factors, such as the presence of antibiotics in the diet or the feeding level, modify cecal microbial communities. It highlights the importance of offering a controlled breeding environment that reduces differences in microbial cecal composition that could be responsible for different animal performance.

## Background

Microbial communities that inhabit the gastrointestinal tract (GIT) of animals constitute a complex ecosystem whose members constantly interact between themselves and with their host [1]. These interactions ensure homeostatic balance maintenance since GIT ecosystem components are involved in many physiological and immunological processes [2]. In the case of the domestic meat rabbit (*Oryctolagus cuniculus*), a small herbivorous mammalian belonging to the family *Leporidae*, cecum is the main organ for microbial fermentation. Thus, it is not surprising that the rabbit cecum hosts the richest and the most diverse microbial community of its GIT [3]. For this reason, the cecum has been the organ preferably chosen in previous rabbit gut microbiota assessments [4–7].

Thanks to the development of next generation sequencing (NGS) technologies, and their rapidly decreasing costs, it is currently possible to characterize the gut microbiota of a large number of animals. This characterization allows a deeper comprehension of the differences between animals concerning their microbial composition and diversity. It is hypothesized that the production environment could partially mediate these differences. Our general aim is to provide further evidence of the effect of different management and environmental factors on cecal microbial composition and diversity. In relation to this topic, there is a certain amount of information already published. A growing number of studies have revealed changes in rabbit cecal microbial communities exerted by age [8] or the type of feed provided to the kits after weaning [6, 7]. Another factor that causes variation is the administration of antibiotics in the feed. Different molecules have been widely administered in rabbit meat production, especially after weaning, to curb mortality peaks (sometimes over 20%) as a result of the onset of gastrointestinal symptoms [9]. Multiple studies have shown alterations caused in gut microbiota by the administration of antibiotics in the feed [5, 10]. Despite the European Union having banned the use of antibiotics in animal feeds as growth promoters since 2006 (EC 1831/2003), at the time this experiment was conducted, the administration of a mix of up to four antibiotics was permitted to prevent or treat the emergence of potential infectious diseases on farms. Nowadays, the administration of only one antibiotic molecule is allowed and substantial efforts are being made towards searching for efficient alternatives which allow for a complete withdrawal of antibiotics in animal feeds. In this context, the application of feed restriction during the growing period was proposed as an interesting alternative to the use of antibiotics. Quantitative feed restriction is a widely applied commercial practice which consists of reducing the amount of feed the animal would consume by a certain percentage when the food is provided ad libitum. Gidenne et al. (2009) [11] demonstrated that feed restriction, despite penalizing animal growth, improves feed efficiency and reduces mortality due to enteric disorders. It is hypothesized that these positive effects could be partially explained by changes in gut microbial composition or activity originated by the application of feed restriction. However, techniques used so far to study this possible association have found no evidence of it [11].

This study, which comprises a large number of animals in an experimental design involving different management and environmental factors, is intended to unravel changes in diversity and composition of rabbit cecal microbial communities associated with these factors. It will allow for a better understanding of how the farm where the animal was raised, the presence of antibiotics in the feed, and feed restriction shape the cecal microbiota of growing rabbits.

## Results

### Sequence processing

**Fig. 1:**
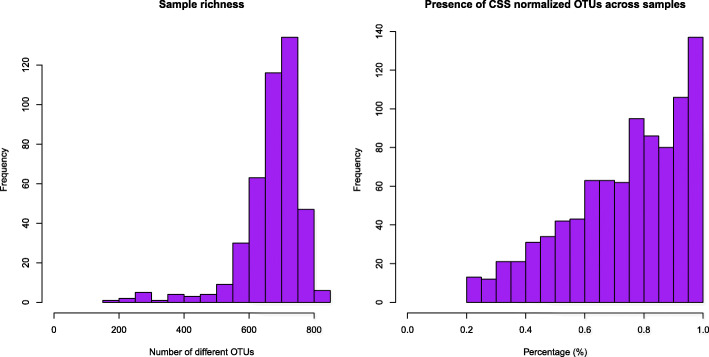
Sample richness and presence of CSS-normalized OTUs across samples

After the removal of doubletons and samples with low sequence counts, 425 rabbit cecal samples (Additional file 1) were represented on 14,928,203 sequence counts clustered into 963 different OTUs. Each sample had on average 35,125 final sequences (range: 10,157-678,798) and 677 OTUs (range: 197–841) (Additional files 2 and 3). Figure 1 shows two histograms representing the sample richness and the proportion of OTUs present across samples. Most of the samples had more than 700 different OTUs (mode = 748) and nearly 140 OTUs were present in all the samples.

Taxonomic assignment of representative OTUs against the Greengenes reference database gg_13_5_otus (Additional file 4) revealed the presence of 8 different known phyla with an average of 8 phyla per sample (range: 7–8) (Additional file 5), and 28 different known genera with an average of 24 genera per sample (range: 17–28) (Additional file 6).

### Animal management and farm environment shaping cecal microbial alpha diversity

**Fig. 2:**
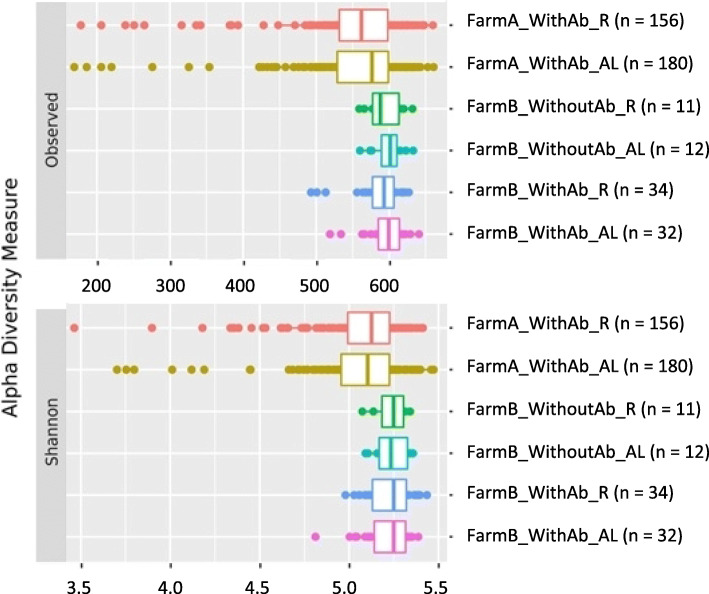
Microbial richness and diversity between samples grouped according to management that animals received. The cecal microbial richness and diversity were estimated by the observed number of different OTUs and the Shannon indexes, respectively

The study of alpha diversity was performed after rarefying the prefiltered and unnormalized OTU table to 10,000 sequences per sample. Rarefaction generated a table which contained the sequence counts of 963 different OTUs for 425 samples. The average (standard deviation) number of observed OTUs within animal was 560.52 (75.03) and the average Shannon index within animal was 5.09 (0.26). The comparison of alpha diversities revealed that the group of animals raised in farm B had greater alpha diversity than the group of animals raised in farm A (estimated differences of 40.20 (9.83) observed OTUs and 0.17 (0.03) Shannon indexes; *P*_*FDR*_ < 0.001). Furthermore, larger variability in both indexes was observed in farm A than in farm B. No significant differences for the two alpha diversity indexes were found between feeding regimes within both farms (Fig. 2, *P*_*FDR*_ > 0.05), nor between the presence and the absence of antibiotics in the feed within farm B (Fig. 2, *P*_*FDR*_ > 0.05).

### Animal management and farm environment shaping cecal microbial composition

**Fig. 3:**
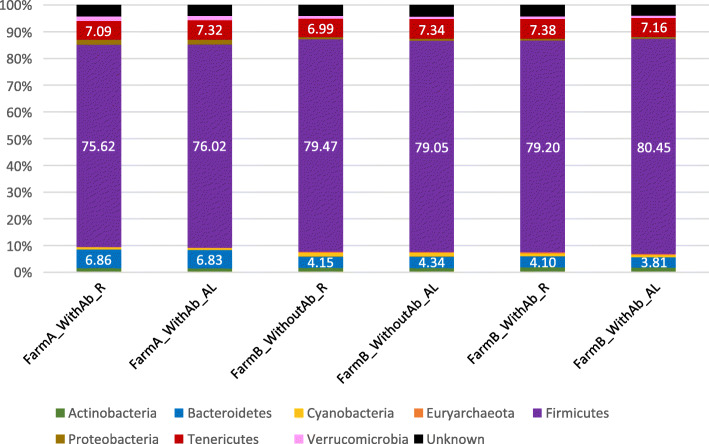
Phyla relative abundances of samples grouped according to farm, level of feeding and presence of antibiotics in the feed

According to the taxonomic assignment of representative sequences (Additional file 4) performed with the UCLUST consensus taxonomy assigner on the Greengenes reference database gg_13_5_otus, *Firmicutes* (76.74%), *Tenericutes* (7.22%) and *Bacteroidetes* (6.26%) were the predominant phyla, accounting for more than 90% of the microbial diversity, in the rabbit cecal samples studied (Fig. 3).

#### Differential growth and cecal microbial composition across farms

The facility where the animals were raised affected their growth performance. Animals raised in farm B exhibited a faster growth (47.11 g/day) than those raised in farm A (44.19 g/day). The estimated average daily gain difference between farm B and farm A was 2.92 ± 0.94 g per day (*P* < 0.005). Cecal samples of rabbits raised in farm A showed an overrepresentation of phyla *Bacteroidetes*, *Proteobacteria* and *Verrucomicrobia* while phyla *Euryarchaeota*, *Cyanobacteria* and *Firmicutes* were found to be overrepresented in cecal samples of rabbits raised in farm B (Table 1).

**Table 1:** Microbial composition at phylum level in cecal samples of rabbits grouped by farm

Phylum	Mean relative abundance in farm A (%) (SD)	Mean relative abundance in farm B (%) (SD)	Estimated difference farm A - farm B ± SE	***P***_**FDR**_
*Actinobacteria*	1.62 (0.67)	1.84 (0.33)	− 0.14 ± 0.08	0.09
*Bacteroidetes*	6.84 (1.81)	4.03 (0.70)	2.74 ± 0.22	0.00
*Cyanobacteria*	0.77 (0.40)	1.05 (0.36)	−0.39 ± 0.05	0.00
*Euryarchaeota*	0.13 (0.19)	0.44 (0.17)	−0.28 ± 0.02	0.00
*Firmicutes*	75.83 (3.34)	79.66 (1.53)	−3.78 ± 0.41	0.00
*Proteobacteria*	1.83 (0.62)	0.66 (0.12)	1.14 ± 0.07	0.00
*Tenericutes*	7.21 (1.47)	7.25 (0.93)	0.00 ± 0.18	0.99
*Verrucomicrobia*	1.62 (0.45)	0.91 (0.24)	0.68 ± 0.05	0.00

Genera *Ruminococcus* (4.32%), *Blautia* (2.96%) and *Oscillospira* (2.37%) dominated the meat rabbit cecal microbiota. Most of the relative abundance differences at genus level were found differentially represented between animals raised in the different farms: genera *Bacteroides*, *Parabacteroides*, *Rikenella*, *Anaerofustis*, *Anaerostipes*, *Clostridium*, *Coprobacillus*, *Anaeroplasma* and *Akkermansia* were overrepresented in cecal samples of rabbits raised in farm A while genera *Adlercreutzia*, *Butyricimonas*, *Odoribacter*, *Methanobrevibacter*, *Blautia*, *Butyrivibrio*, *Coprococcus*, *Dehalobacterium*, *Dorea*, *Oscillospira*, *rc4–4* and *Oxalabacter* were overrepresented in cecal samples of rabbits raised in farm B. Interestingly, genera *Epulopiscium*, *p-75-a5*, *Phascolarctobacterium*, *Campylobacter* and *Desulfovibrio* were only found in samples collected from farm A (Table 2).

**Table 2:** Relative abundances of genera, grouped by phylum, differentially represented between farms (*P*_*FDR*_ < 0.05)

Genus	Mean relative abundance in farm A (%) (SD)	Mean relative abundance in farm B (%) (SD)	Estimated difference farm A - farm B ± SE
***Actinobacteria***			
*Adlercreutzia*	0.89 (0.47)	1.14 (0.23)	−0.19 ± 0.06
***Bacteroidetes***			
*Bacteroides*	1.88 (0.67)	0.80 (0.35)	1.10 ± 0.08
*Butyricimonas*	0.16 (0.19)	0.35 (0.17)	−0.19 ± 0.02
*Odoribacter*	0.23 (0.21)	0.44 (0.20)	−0.21 ± 0.03
*Parabacteroides*	0.25 (0.18)	0.07 (0.07)	0.18 ± 0.02
*Rikenella*	0.39 (0.24)	0.18 (0.13)	0.25 ± 0.03
***Euryarchaeota***			
*Methanobrevibacter*	0.13 (0.19)	0.44 (0.17)	−0.28 ± 0.02
***Firmicutes***			
*Anaerofustis*	0.12 (0.08)	0.08 (0.04)	0.03 ± 0.01
*Anaerostipes*	0.17 (0.08)	0.12 (0.04)	0.06 ± 0.01
*Blautia*	2.86 (0.67)	3.22 (0.46)	−0.36 ± 0.08
*Butyrivibrio*	0.10 (0.07)	0.13 (0.06)	−0.03 ± 0.01
*Clostridium*	1.09 (0.26)	0.87 (0.13)	0.21 ± 0.03
*Coprobacillus*	0.20 (0.27)	0.14 (0.08)	0.08 ± 0.03
*Coprococcus*	1.96 (0.42)	2.26 (0.29)	−0.28 ± 0.05
*Dehalobacterium*	0.05 (0.08)	0.18 (0.03)	−0.13 ± 0.01
*Dorea*	0.46 (0.12)	0.51 (0.09)	−0.05 ± 0.02
*Epulopiscium*	0.14 (0.11)	0.00 (0.00)	0.15 ± 0.01
*Oscillospira*	2.11 (0.53)	2.85 (0.31)	−0.79 ± 0.07
*p-75-a5*	0.13 (0.06)	0.00 (0.00)	0.13 ± 0.01
*Phascolarctobacterium*	0.27 (0.24)	0.00 (0.00)	0.26 ± 0.03
*rc4–4*	0.13 (0.06)	0.23 (0.03)	−0.10 ± 0.01
***Proteobacteria***			
*Campylobacter*	0.08 (0.08)	0.00 (0.00)	0.08 ± 0.01
*Desulfovibrio*	0.58 (0.22)	0.00 (0.00)	0.57 ± 0.03
*Oxalabacter*	0.10 (0.06)	0.13 (0.03)	−0.03 ± 0.01
***Tenericutes***			
*Anaeroplasma*	0.23 (0.18)	0.10 (0.09)	0.12 ± 0.02
***Verrucomicrobia***			
*Akkermansia*	1.62 (0.45)	0.91 (0.23)	0.68 ± 0.05

The analyses on the CSS-normalized OTUs revealed that 648 out of the 946 OTUs showed signatures significantly different between farms. Out of these, 276 were overrepresented in farm A, while 372 were overrepresented in farm B. Table S1 shows the estimated difference between farms for these OTUs, their sequences and their assignment at the lowest taxonomic level. Only 9 of them could be assigned at species level and 129 were assigned to known genera. These results showed remarkable coincidences with those obtained from the analyses directly performed on the relative abundance of taxa at phylum and genera levels. An example that illustrates this match is the overrepresentation of genus *Akkermansia* in farm A. This genus is encompassed by phylum *Verrucomicrobia* that was also overrepresented in rabbits raised in farm A, as well as 6 out of the 7 OTUs assigned to this phylum.

#### Differential growth and cecal microbial composition across feeding regimes

The feeding regime affected the rabbits’ growth performance in both facilities. Animals fed AL had a higher growth (48.74 and 55.77 g/day in farms A and B, respectively) than those fed R (38.95 and 38.65 g/day in farms A and B, respectively). The estimated average daily gain difference between AL and R groups was 9.79 ± 0.58 and 17.12 ± 1.08 g per day in farms A and B, respectively (*P* < 0.001). An overrepresentation of phyla *Cyanobacteria* (estimated difference R - AL = 0.11 ± 0.04; *P*_*FDR*_ = 0.04) and *Verrucomicrobia* (estimated difference R - AL = 0.11 ± 0.05; *P*_*FDR*_ = 0.04) was found in cecal samples of rabbits fed R and raised in farm A. On the other hand, phylum *Euryarchaeota* was overrepresented in animals fed R and raised in farm B (estimated difference R - AL = 0.14 ± 0.04; *P*_*FDR*_ < 0.001). At genus level, the only significant contrast was observed for *rc4–4* which resulted overrepresented in samples from animals fed AL in farm A (estimated difference R - AL = − 0.03 ± 0.01; *P*_*FDR*_ < 0.001) while in farm B none of the genera resulted differentially represented (*P*_*FDR*_ > 0.05) between feeding regimes. The contrasts based on the CSS-normalized OTUs revealed 51 and 9 OTUs differentially represented between feeding regimes within farms A and B, respectively. Within farm A, 32 OTUs were overrepresented in cecal samples of rabbits that were fed AL and 19 OTUs in the samples from rabbits fed R. Within farm B, 7 OTUs were overrepresented in cecal samples of rabbits that were fed AL and 2 OTUs were overrepresented in rabbits that were fed R. Table S2 shows the estimated difference between feeding regime within farm of these OTUs, their sequences and their assignment at the lowest taxonomic level. The analyses based on the CSS-normalized OTUs within farm A were in full accordance with the analyses performed at genus level given that all OTUs assigned to genus *rc4–4* (phylum *Firmicutes*) were overrepresented in cecal samples of rabbits fed AL.

#### Effect of the presence of antibiotics in the feed

The effect of the presence of antibiotics in the feed could only be assessed within farm B given that all rabbits raised in farm A received feed supplemented with antibiotics. Animals that received antibiotics had a slightly higher growth (47.29 g/day) than those that did not (46.59 g/day). The estimated average daily gain difference between groups was not significant (0.69 ± 2.43 g per day; *P* = 0.78). Cecal samples of rabbits that received feed free of antibiotics showed an overrepresentation of phyla *Cyanobacteria* compared to those that received feed supplemented with antibiotics (estimated difference without antibiotics - with antibiotics = 0.49 ± 0.09; *P*_*FDR*_ < 0.001). In addition, the analyses on the CSS-normalized OTUs revealed an overrepresentation of 15 and 29 OTUs in cecal samples of rabbits that received a feed supplemented or free of antibiotics; respectively. Table S3 shows the estimated difference between the presence and the absence of antibiotics in the feed for the OTUs in which the differences reached the significance threshold. The OTU sequences as well as their assignment at the lowest taxonomic level are also shown in Table S3. Only 1 of these OTUs could be assigned at species level (*Bacteroides fragilis*) and 2 OTUs at genus level (*Oscillospira* and *Coprococcus*).

### Microbial information as a classifier of cecal samples according to farm environment and animal management

**Fig. 4:**
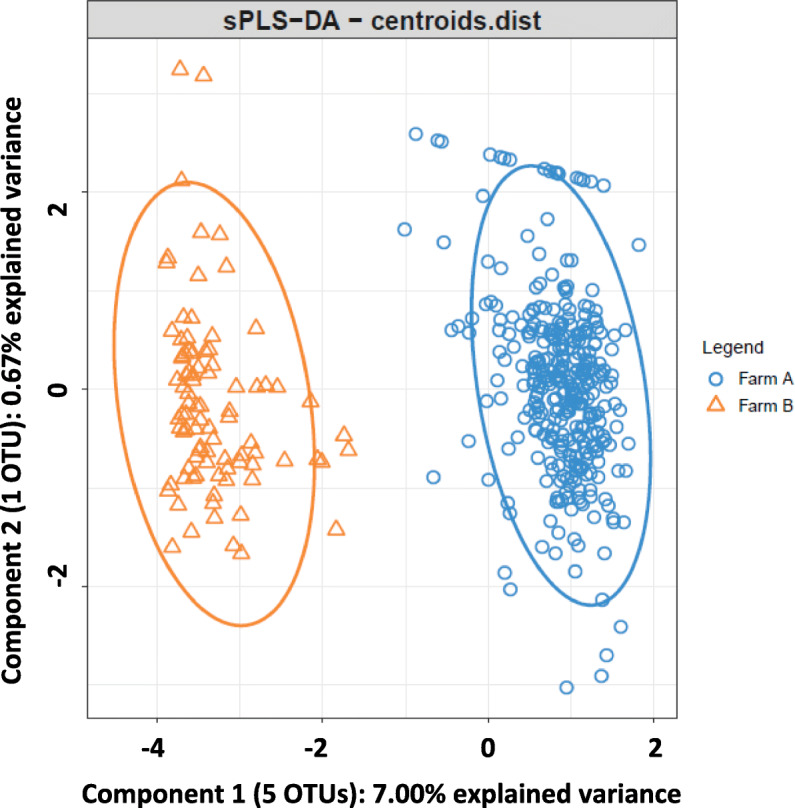
Sparse partial least squares discriminant analysis representing cecal samples of rabbits raised in farm A (blue) and in farm B (orange)

Sparse partial least squares-discriminant analyses (sPLS-DA) on the CSS-normalized OTUs were conducted to discriminate samples according to the factors considered in this study (i.e., the farm where the animal was raised, the presence or the absence of antibiotics in the feed and the feeding regime). The tuning process of the sPLS-DA conducted to discriminate samples according to the farm where the rabbits were raised selected 5 OTUs for component 1 and 1 OTU for component 2 (Fig. 4). Component 1 explained 7.00% of the total variance while component 2 explained 0.67%. The classification performance of this sPLS-DA could be said to be perfect since its overall and balanced error rate (BER) per class across 1000 replicates of 5-folds cross-validation runs was 0.00 (0.00). Furthermore, two OTUs of component 1 had a stability higher than 0.9.

**Fig. 5:**
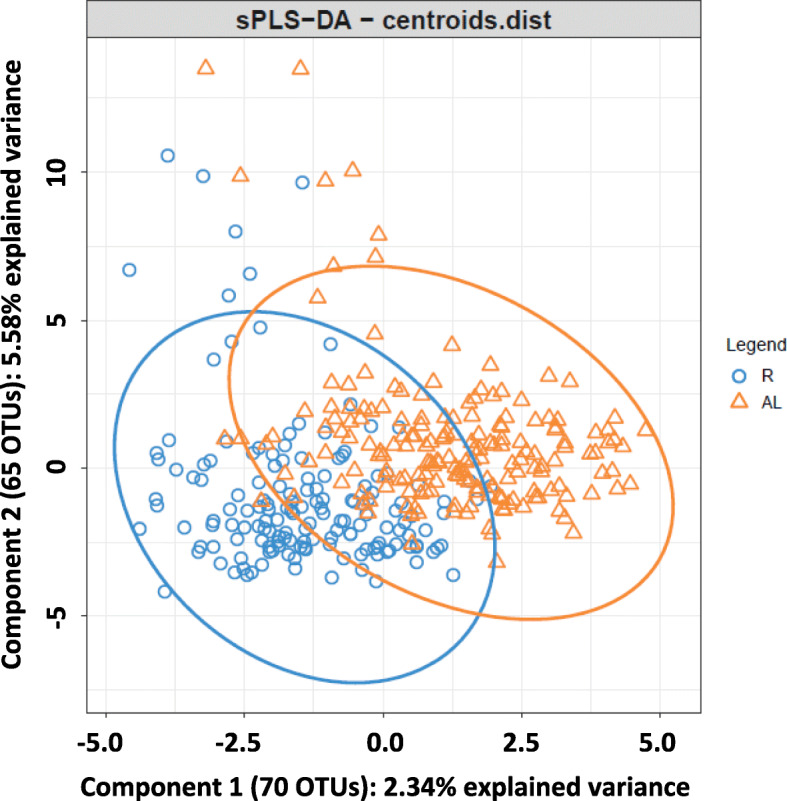
Sparse partial least squares discriminant analysis representing cecal samples of rabbits raised in farm A and fed R (blue) or AL (orange)

The sPLS-DA performed to discriminate samples across feeding regimes within farm A selected 70 OTUs for component 1 and 65 OTUs for component 2 (Fig. 5). Component 1 explained 2.34% of the total variance while component 2 explained 5.58%. The cross-validation assessment of the classification performance of this sPLS-DA showed an overall and BER per class of 0.27 (0.02). The stability of 18 and 5 OTUs selected in components 1 and 2, respectively, across the different cross-validation folds was higher than 0.9.

**Fig. 6:**
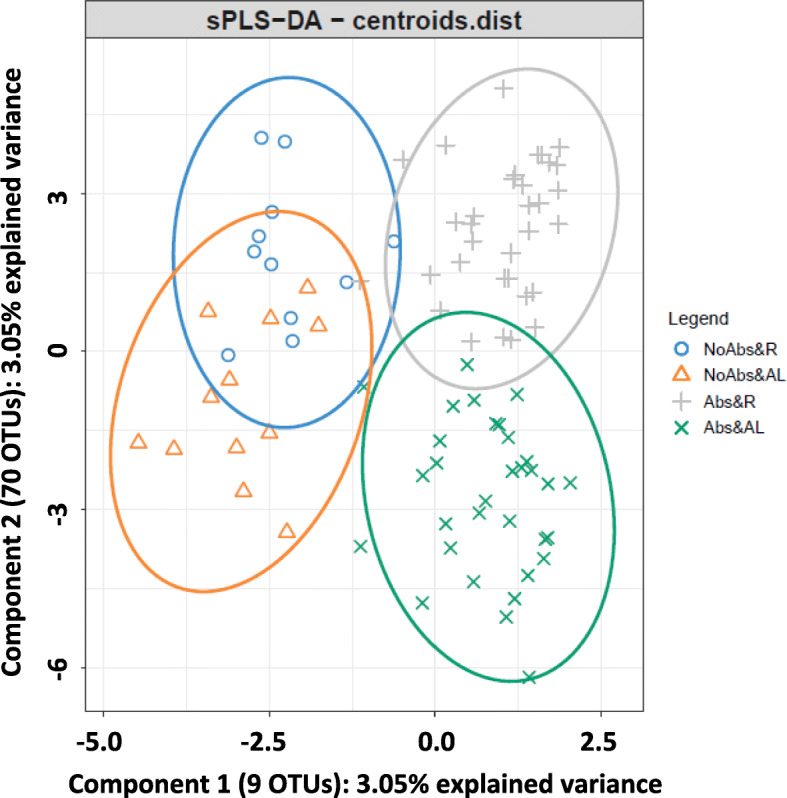
Sparse partial least squares discriminant analysis representing cecal samples of rabbits raised in farm B and fed R without antibiotics (blue), fed AL without antibiotics (orange), fed R with antibiotics (gray) and fed AL with antibiotics (green)

Finally, the sPLS-DA conducted to discriminate samples of animals raised within farm B according to the combination of the presence or not of antibiotics in the feed and the feeding regime selected 9 OTUs for component 1 and 70 OTUs for component 2 (Fig. 6). Component 1 explained 3.05% of total variance and defined the discrimination between samples from animals fed with antibiotics and those fed without antibiotics. On the other hand, component 2 explained 3.05% of total variance and defined the discrimination between samples from animals fed R and those belonging to animals fed AL. The cross-validation assessment of the classification performance of this sPLS-DA showed an overall BER of 0.32 (0.15). The BER per class was 0.34 (0.12) for samples fed R without antibiotics, 0.46 (0.14) for samples fed AL without antibiotics, 0.29 (0.11) for samples fed R with antibiotics, and 0.20 (0.07) for samples fed AL with antibiotics. The stability of 3 and 11 OTUs selected in components 1 and 2, respectively, across the different cross-validation folds was higher than 0.9.

## Discussion

The influences of farm environment and common commercial practices of animal management on their gut microbiota are not yet well known in many livestock species. In this study, we have aimed to disentangle potential changes in microbial diversity and composition of meat rabbit cecal communities as a result of being raised in different farms and subjected to different handling during their growing period. To shed light on this matter, we conducted a microbiota comparison of a large number of rabbits raised in different farms, feeding regimes, and fed with feed supplemented or free of antibiotics.

### 16S rRNA gene-based characterization of meat rabbit cecal microbiota

The Illumina MiSeq sequence processing of samples collected from these animals revealed that phyla *Firmicutes*, *Tenericutes* and *Bacteroidetes* dominate the growing meat rabbit cecal ecosystem representing more than 90% of its entire microbial composition. This fact is in accordance with previous studies that have characterized the rabbit cecal microbiota [5, 7, 12] and reported *Firmicutes* as the predominant phylum. However, there are discrepancies between studies in establishing which other phyla are also prevalent in this ecosystem. Whereas we found phyla *Tenericutes* and *Bacteroidetes* representing 7.22 and 5.93% of the cecal microbial composition, respectively, Chen et al. 2019 [7] and Zou et al. 2016 [5] reported *Bacteroidetes* as the second predominant phylum representing 18 and 20% of New Zealand White and Rex rabbit cecal microbial composition, respectively. Conversely, other studies that have previously characterized meat rabbit fecal microbiota identified higher relative abundances of phyla *Proteobacteria* and *Verrucomicrobia* [10, 13]. Velasco-Galilea et al. 2018 [12] reported *Firmicutes* (76.42%), *Tenericutes* (7.83%) and *Bacteroidetes* (7.42%) as the predominant phyla of meat rabbit fecal and cecal microbial communities. These discrepancies found across studies could be attributed to technical issues (e.g., pair of primers, sequencing platform, bioinformatic pipeline employed to process raw sequences or reference database used for the taxonomic assignment of the representative sequences) or to purely biological reasons (e.g., breed, age or section of the GIT sampled). Nonetheless, Kylie et al. (2018) [13] depicted that the relative increase in less beneficial phyla, such as *Proteobacteria*, could be related to seasonal climate changes that directly impact rabbits’ health. This impact affects the susceptibility to enteritis and possibly feed conversion efficiency. In any case, this phylum was more prevalent in farm A where the animals were more exposed to changes in climate conditions.

### Farm environment modify alpha diversity

Regarding the alpha diversity assessment, Shannon and the observed number of OTUs indexes revealed the existence of significant differences between the experimental farm where the rabbits were raised. Cecal samples collected from rabbits raised in farm B had greater richness and diversity than those belonging to animals raised in farm A. This could be explained by more stable environmental conditions in farm B (i.e., facility better insulated) than in farm A. It has been already shown that intestinal health is positively associated with microbial diversity [14]. In our case, this better health could be said to be granted by the more stable environmental conditions offered by farm B. The most exposed environmental conditions of farm A, combined with the fact that samples of animals raised in this facility were collected from rabbits produced in 4 different batches, could also explain the larger variability in both indexes observed in this farm [13]. Despite not having observed significant differences between the presence or not of antibiotic in the feed, nor between feeding regimes, it is noteworthy to mention that samples collected from animals fed AL in both farms had a greater, although not significant, richness than those fed R. This fact is consistent with previous studies in mice that observed a lower alpha diversity in animals with a restricted level of feeding [15–17]. Surprisingly, but in agreement with our results, studies performed in pigs [18], chicken [19] and Rex rabbits [5] also did not show clear significant differences on alpha diversity indexes between animals fed on diets with antibiotics with respect to those on diets free of antibiotics. Nevertheless, these studies were able to detect differences in the relative abundances of some specific species between diets. For example, Kumar et al. 2018 [19] found that the inclusion of bacitracin in the feed did not affect the chicken bacterial phyla. However, they observed differences between the control and the bacitracin-fed group in the ileal and cecal bacterial populations at lower taxonomic levels. It is worth noting that the antibiotic withdrawal at the beginning of the last week of the rabbits’ lives equalized the diets of both groups and possibly their microbial populations, which may explain some lack of differences between them.

### Farm environment has a large impact on rabbit cecal microbiota

Despite the lack of differences in microbial diversity and richness across management factors; univariate studies revealed differential microbial composition across the studied factors. In addition, the performed multivariate analysis evidenced a certain classification power of the samples on the different levels of management and environment factors based on the microbial composition of the samples.

As it might be expected, analyses of variance confirmed that the breeding farm strongly impacts meat rabbit cecal microbial composition. Our results revealed that the relative abundances of 6 out of 8 phyla are differentially represented between both farms. At genus level, we detected significant differences in the relative abundances of almost all of them. Genera *Bacteroides*, *Parabacteroides*, *Rikenella*, *Anaerofustis*, *Anaerostipes*, *Clostridium*, *Coprobacillus*, *Anaeroplasma* and *Akkermansia* were enriched in cecal samples of rabbits housed in farm A. The first three belong to phylum *Bacteroidetes* and genus *Bacteroides* is the most abundant of them in meat rabbit cecum. Species of this genus are anaerobic Gram-negative members of the family *Bacteroidaceae* that play an important role in the degradation of vegetal polysaccharides and amino acid fermentation in the mammal GIT [20, 21]. Moreover, this genus is involved in propionic acid and lactate formation depending on nitrogen organic availability. Nonetheless, some authors showed that great amounts of *Bacteroides* could predict obesity tendency. *Parabacteroides* is also an anaerobic Gram-negative bacterium (family *Porphyromonadaceae*) involved in amino acid transport and metabolism, energy production and conversion, lipid transport and metabolism, recombination and repair, cell cycle control, cell division, and cell motility in the intestinal microbiota of the growing rabbit [22]. This genus was specifically found in the cecal microbiota of mice raised in conventional conditions and absent in those raised in pathogen-free facilities in a study performed under different housing conditions [23].

Within the phylum *Firmicutes*, genus *Clostridium* (family *Clostridiaceae*) is an anaerobic Gram-positive bacterium that inhabits the GIT of many mammals where it acts by degrading cellulose. However, some *Clostridium* species (e.g., *C. perfringens* and *C. difficile*) are pathogenic, and an enrichment of this genus has previously been described in rabbits affected by epizootic rabbit enteropathy [24]. This genus, together with genus *Bacteroides*, was found enriched in the cecal microbiota of mice housed in open cages compared with those kept in individual ventilated cages [25]. Both genera have been associated with an exacerbation of the intestinal inflammatory response in mammals [26]. Genus *Anaerofustis* (family *Eubacteriaceae*) has been found enriched in cecal samples of rabbits affected by paratuberculosis infection (*Mycobacterium avium*) [27].

Within the phylum *Verrucomicrobia*, genus *Akkermansia* is an anaerobic Gram-negative bacterium that encompasses mucin degrader species [28]. In the cecum, a proper enrichment of this genus could maintain a suitable mucosal turn-over, thus exerting a protective effect that could help the animal to deal with inflammatory processes.

It is worth mentioning that we have detected genera *Epulopiscium*, *p-75-a5*, *Phascolarctobacterium*, *Campylobacter* and *Desulfovibrio* only in the cecal samples of rabbits housed in farm A. The first three are encompassed within the phylum *Firmicutes.* Genus *Epulopiscium* is a large size Gram-positive bacterium that has a nutritional symbiotic relationship with surgeonfish that eats algae and detritus. This bacterium is physically similar to the phylogenetically related *Metabacterium polyspora* which is an endospore-producing bacterium isolated from the cecum of guinea pigs [29]. On the other hand, genera *Campylobacter* and *Desulfovibrio* are Gram-negative bacteria that belong to phylum *Proteobacteria*. Some species of these genera are pathogens responsible for infections and diarrheas in mammals. The exclusive presence of these genera in farm A could indicate the existence of a potential dysbiosis of the animals raised in that facility that could affect their sanitary status and growth. While farm A was a semi-open-air facility, farm B was artificially ventilated and offered more controlled environmental conditions that favor animal growth. Moreover, the presence of sulfate-reducing bacteria (SRB) such as *Desulfovibrio* could be enhanced by sulfate-secreting bacteria (SSB) such as *Rikenella* in farm A where this genus is significantly more predominant. It is noteworthy to mention that SRB could also obtain sulfate via *cross-feeding* mediated by *Bacteroides*-encoded sulfatases [30], and interestingly, this phylum is more prevalent in farm A.

Regarding sample classification based on the sPLS-DA study, given the important differences in gut microbial composition found between farms, a perfect classification of the samples can be achieved with only 5 OTUs. One of these 5 OTUs was overrepresented in farm B and belonged to family *S24–7* (phylum *Bacteroidetes*). The remaining 4 were overrepresented in farm A and belonged to family *Barnesiellaceae* (phylum *Bacteroidetes*), order *Bacteroidales* (phylum *Bacteroidetes*), and genera *Desulfovibrio* (phylum *Proteobacteria*) and *Bacteroides* (phylum *Bacteroidetes*). It is worth mentioning that these 5 OTUs were also declared as differentially represented between farms by the univariate analyses.

### Administration of antibiotics impact on some taxa relative abundances

Within farm B, the effect of the presence of antibiotics in the feed was assessed by comparing the microbial cecal composition of rabbits fed with antibiotics with that of some animals that received feed without antibiotics. As stated above, we did not detect significant differences in alpha diversity, nor in genera relative abundances, between both groups. However, some significant differences were observed at phylum and OTU levels. An overrepresentation of phylum *Cyanobacteria* was found in rabbits fed without antibiotics. The detection of this bacterial phylotype, commonly assigned to photosynthetic activity, in the rabbit cecum could suggest contamination during the GIT sampling. However, Zeng et al. 2015 [31] previously reported its presence in rabbit feces. In the present study, all OTUs taxonomically assigned to phylum *Cyanobacteria* are as well encompassed in the order *YS2*. Interestingly, it was demonstrated that this order does not really have photosynthetic capacity and it is currently classified within the candidate phylum *Melainabacteria* [32]. The non-photosynthetic cyanobacteria *YS2*, now named *Gastranaerophilales*, is a fermenter gut-associated order present in humans and other animals such as squirrels, where its exact role is unknown but it has the capacity to produce hydrogen, fix nitrogen and synthesize vitamins B and K [32–34]. Our results, in accordance with Kylie et al. 2018 [13], revealed that rabbits fed without antibiotics exhibited higher abundances of OTUs assigned to phylum *Bacteroidetes* than those fed with antibiotics. In addition, samples of rabbits that received antibiotics had a significant increase of an OTU taxonomically assigned to genus *Coprococcus.* Interestingly, a study that evaluated the differences in bacterial communities of Rex rabbits fed with different antibiotics also found an overrepresentation of this bacterium in animals treated with zinc bacitracin [5]. *Coprococcus* is an anaerobic bacterium that may protect against colon cancer in humans by producing butyric acid [35]. We hypothesized that the administration of antibiotics could modulate the abundance of some *Coprococcus* species to provide intestinal protection on meat rabbits. However, it is important to recognize that the reduced sample size of the group of rabbits fed without antibiotics may have limited the statistical power to detect microbial composition differences associated with this factor.

### Feed restriction modify *Euryarchaeota* and some bacteria relative abundances

Within farm B, the effect of the feeding regime in microbial composition was also assessed by comparing samples of animals fed R with those fed AL. The main difference found was for phylum *Euryarchaeota* which was overrepresented in animals fed R in farm B. All *Euryarchaeota* species found in the rabbit cecum belong to genus *Methanobrevibacter* that encompasses different hydrogenotrophic methane-producing species. Previous studies in humans [36] and cattle [37, 38] found an overrepresentation of *Methanobrevibacter* species in individuals submitted to feed restriction and a negative correlation between the abundance of this bacterium and body mass index. A prevalence of *Methanobrevibacter* species could be a positive indicator of a healthy microbiota since restricted animals showed an overrepresentation of this genus. The main purpose of applying feed restriction is to improve intestinal health, reducing weaning mortality. The growth of *Methanobrevibacter* is supported by fermenters such as *Gastranaerophilales* and butyrate-producing bacteria such as *Anaereostipes* via interspecies formate/hydrogen transfer [39]. A study in mice determined that *Methanobrevibacter smithii* facilitates *Bacteroides thetaiotaomicron* capacity to digest glycans resulting in increased production of short-chain fatty acids [40]. The same study defined *M. smithii* as a “*power broker*” that regulates polysaccharide fermentation efficiency that influences the fat stores. The lower prevalence of methanogenic archaea in farm A could be explained by the high presence of SRB that outcompete with methanogens for hydrogen consumption. This fact could favor hydrogen sulfide production and compromise the rabbits’ health.

Regarding the sample classification based on the sPLS-DA study conducted within farm B, component 1 and component 2 discriminated between animals that did or did not received antibiotics in the feed and between feeding regimes, respectively. It is worth mentioning that 8 out of 9 OTUs selected in component 1 were also declared as differentially represented between the presence or the absence of antibiotics in the feed by the univariate analyses. Within farm A, an sPLS-DA was also performed to classify samples according to the feeding regime using microbial information. Although a large number of OTUs were selected as classifier variables in the tuning process of this sPLS-DA, the classification error rate was high. It implied a poor discrimination capacity of samples according to the feeding regime the animal received. Nevertheless, bootstrap univariate analyses of variance detected some significant differences at all taxonomic levels analyzed between feeding regimes within farm A. At genus level, *rc4–4* was overrepresented in animals fed AL. This genus belongs to phylum *Firmicutes* and it is known as an obesity-associated bacterium [41] and as a pathogenic candidate identified in mice with multiple sclerosis [42]. A potential pro-inflammatory role has been proposed for this genus [42] what could be related to a reduced incidence of enteric disorders when feed restriction is applied. It is worth mentioning that family *Peptococcaceae*, which encompasses genus *rc4–4*, is strongly related to total rabbit weight gain from weaning to 12-week old [43]. Although in our study this genus was prevalent in animals fed AL, its association with weight gain is not clear since the greater growth exhibited by these animals was consequence of higher feed intake.

### Rabbit cecal microbiota is shaped by farm environment and animal management

Different approaches have been applied in this study to evaluate the effect of different environments and management practices, commonly used in rabbit production, in their cecal microbial composition and diversity. Those animals raised in the best insulated facility (farm B) appear to have a microbiota characteristic of healthier animals than those raised in the open-air facility (farm A). It is worth mentioning that the rabbits were housed in cages interspersed with feeding regime. This fact could make possible the exchange of microorganisms between animals of different feeding regimes and therefore have reduced the differences observed between regimes. However, the joint consideration of 70 OTUs in the sPLS-DA made possible a certain discrimination power of samples according to the level of feeding received by each animal raised in farm A. It implies the existence of cecal microbiota content patterns characteristic of each regime which could be revealed thanks to the univariate analyses conducted at different taxonomic levels. Similarly, the sPLS-DA performed within farm B also involved the consideration of 70 OTUs to discriminate samples according to the amount of feed consumed. Within this farm, the classification of samples regarding the presence or the absence of antibiotics in the feed needed a smaller number of OTUs than the feeding regime but greater than the farm. This suggests that the effect of the presence of antibiotic in feed is stronger than the feeding level. The lack of a group of samples collected from animals that did not receive antibiotics precluded the evaluation of the magnitude of importance of this effect over the feeding level on the cecal microbiota of animals raised farm A. It might have been possible that the magnitude of the effect of the presence of antibiotics in the feed was larger in farm A than that observed in farm B. The experimental design of this study prevented the comparison of the effect of antibiotic treatments across farms on rabbits’ microbial communities. The implication of the discussed microbial composition and diversity differences originated by the studied management and environmental factors on the animals’ performance still needs to be investigated. In future studies the role of specific groups of bacteria in rabbit growth and feed efficiency will be analyzed.

## Conclusions

The analysis of a large number of animals from a paternal rabbit line has allowed a deeper comprehension of the role played by different management and environmental factors shaping the composition and diversity of cecal microbial communities. It reveals that the farm environment offered to the rabbits during their growth play a key role that can result in different microbial alpha diversity and composition of almost all species that inhabit the rabbit GIT. This highlights the importance that a stable and controlled environment could have in the intestinal health and, consequently, in animal performance. It seems clear that the better insulated conditions of farm B favored the presence of a gut microbiota characteristic of healthier animals. Although the level of feeding and the presence of antibiotics in the feed did not modify the global diversity of cecal microbial communities, these factors can increase or decrease the prevalence of specific bacteria which could lead to a microbial composition potentially beneficial for the animal or, at the other extreme, to an origin of future intestinal dysbiosis.

## Methods

### Animals and experimental design

All biological samples used in the study were collected from animals of an experiment conducted at the Institute of Agrifood Research and Technology (IRTA) in different periods and involving two different farms. The objective of that experiment was to estimate the effect of the interaction between the genotype and the feeding regime (i.e., the amount of feed provided during fattening) on growth, feed efficiency, carcass characteristics, and health status of the animals [44]. For this particular study, 425 meat rabbits from Caldes line [45] of that experiment were randomly selected. Most of them (336) were raised in 4 different batches in a semi-open-air facility (farm A). The remaining animals (89) were produced in a single batch in another facility under better controlled environmental conditions (farm B). Rabbits raised in farm A were housed in collective cages containing 8 kits each one while those raised in farm B were housed in cages with 6 kits each one. All animals were raised under the same management conditions and received the same standard pelleted diet. Twenty-three rabbits raised in farm B received a diet free of antibiotics and the remaining sixty-six received the same diet but supplemented with antibiotics. Those raised in farm A received oxytetracycline, valnemulin, and colistin while those in farm B received oxytetracycline, valnemulin and neomycin. At the time this experiment was conducted, it was possible to use up to four types of molecules to prevent or treat the emergence of potential infectious diseases on farms. However, nowadays, only one antibiotic molecule is allowed. During the last fattening week all the animals received an antibiotic free diet. Feed was supplied once per day in a feeder with three places for the 4–5 weeks that the fattening lasted. Water was provided ad libitum during the whole fattening period. The animals were under two different feeding regimes: (1) ad libitum (AL) or (2) restricted (R) to 75% of the AL feed intake. The amount of feed supplied to the animals under R feeding regime in a given week for each batch was computed as 0.75 times the average feed intake of kits on AL from the same batch during the previous week, plus 10% to account for a feed intake increase as the animal grows. Kits were randomly assigned to one of these two feeding regimes after weaning (32 days of age). They were categorized into two groups according to their size at weaning (big if their body weight was greater than 700 g or small otherwise) aiming to obtain homogenous groups regarding animal size within feeding regime. A maximum of two kits of the same litter were assigned to the same cage in order to remove the possible association between cage and maternal effects on animal growth during the fattening period. The distribution of these animals across the different levels of management factors is shown in Table 3. The body weight of each animal was weekly recorded. The individual average daily gain was computed as the slope of the within animal regression of all body weight measurements recorded during the growing period.

**Table 3:** Distribution of rabbits in groups according to different management factors

Farm	Batch	Feed	Feeding regime	Number of rabbits
A	1	With antibiotics	Ad libitum	27
A	1	With antibiotics	Restricted	30
A	2	With antibiotics	Ad libitum	35
A	2	With antibiotics	Restricted	41
A	3	With antibiotics	Ad libitum	61
A	3	With antibiotics	Restricted	53
A	4	With antibiotics	Ad libitum	57
A	4	With antibiotics	Restricted	32
B	5	With antibiotics	Ad libitum	32
B	5	With antibiotics	Restricted	34
B	5	Without antibiotics	Ad libitum	12
B	5	Without antibiotics	Restricted	11

### Sample processing, DNA extraction and sequencing

Animals were slaughtered (at 66 and 60 days of age in farm A and farm B, respectively) and cecal sample of each rabbit were collected in a sterile tube, kept cold in the laboratory (4 °C) and stored at − 80 °C. DNA extraction, amplification, Illumina library preparation and sequencing followed methods described previously [12]. Whole genomic DNA was extracted from 250 mg of each cecal samples using ZR Soil Microbe DNA MiniPrep™ kit (ZymoResearch, Freiburg, Germany) according to manufacturer’s instructions with the following modification: cecal samples were mechanically lysed in a FastPrep-24™ Homogenizer (MP Biomedicals, LLC, Santa Ana, CA, United States) at a speed of 1 × 6 m/s for 60 s facilitating an efficient lysis of archaea and bacteria species. Integrity and purity of DNA extracts were measured with Nanodrop ND-1000 spectrophotometer equipment (NanoDrop products; Wilmington, DE, United States) according to Desjardins and Conklin’s protocol [46]. All DNA extracts had adequate integrity and purity (absorbance ratio 260 nm/280 nm > 1.6) to avoid PCR inhibition issues.

A fragment of the 16S rRNA gene including the V4-V5 hypervariable regions was amplified with F515Y/R926 primer combination (5′-GTGYCAGCMGCCGCGGTAA-3′, 5′-CCGYCAATTYMTTTRAGTTT-3′) [47] and then re-amplified in a limited-cycle PCR reaction to add sequencing adaptors and 8 nucleotide dual-indexed barcodes of multiplex Nextera® XT kit (Illumina, Inc., San Diego CA, United States) following manufacturer’s instructions. The initial PCR reactions were performed for each sample using 12.5 μl 2x KAPA HiFi HotStart Ready Mix, 5 μl forward primer, 5 μl reverse primer and 2.5 μl template DNA (5 ng/ μl). The initial PCR conditions were as follows: initial denaturation for 3 min at 95 °C, 25 cycles of 30 s at 95 °C, 30 s at 55 °C and 30 s at 72 °C; and final extension for 2 min at 72 °C. The addition of indexes and sequencing adaptors to both ends of the amplified regions took place in a second PCR by using 25 μl 2x KAPA HiFi HotStart Ready Mix, 5 μl index i7, 5 μl index i5, 10 μl PCR Grade water and 5 μl concentrated amplicons of initial PCR. The second PCR conditions were as follows: initial denaturation for 3 min at 95 °C, 8 cycles of 30 s at 95 °C, 30 s at 55 °C and 30 s at 72 °C; and final extension for 5 min at 72 °C. Final libraries were cleaned up with AMPure XP beads, validated by running 1 μl of a 1:50 dilution on a Bioanalyzer DNA 1000 chip (Agilent Technologies, Inc., Santa Clara, CA, United States) to verify their size, quantified by fluorometry with PicoGreen dsDNA quantification kit (Invitrogen, Life Technologies, Carlsbad, CA, United States), pooled at equimolar concentrations and paired-end sequenced in 5 parallel plates in a Illumina MiSeq 2 × 250 platform at the Genomics and Bioinformatics Service (SGB) of the Autonomous University of Barcelona (UAB).

### Bioinformatic pipeline for OTU calling

Sequence processing was performed using QIIME software (version 1.9.0) [48]. In a first step, the resulting paired-ended V4-V5 16S rRNA gene reads were assembled into contigs with the python script *multiple_join_paired_ends.py*. Then the contigs were curated using the script *split_libraries.py* with default parameters in order to assign them to samples and to discard those with a low-quality (Q19 was the minimum acceptable quality score). Chimeric sequences generated during the process of DNA amplification were detected with a UCHIME algorithm [49] and removed. The totality of filtered contigs were clustered into operational taxonomic units (OTUs) with a 97% similarity threshold using the script *pick_open_reference_otus.py* with default parameters [50] that grouped, through a UCLUST algorithm [51], the sequences against Greengenes reference database (version gg_13_5_otus) and also made a de novo clustering of those that did not match the database. The generated OTU table was filtered at: (1) sample level: by discarding samples with less than 5000 final sequence counts and at (2) OTU level: by removing the doubleton ones. The filtered OTU table contained the sequence counts of 963 OTUs for 425 samples. Taxonomic assignment of representative sequences of each OTU defined (963) was conducted by mapping them to the Greengenes reference database gg_13_5_otus with the UCLUST consensus taxonomy assigner (QIIME default parameters). The raw sequence data were deposited in the sequence read archive of NCBI under the BioProject accession number PRJNA524130. Metadata, the prefiltered and normalized OTU tables, and corresponding taxonomic classifications are also included as Additional files 1, 2, 3 and 4, respectively.

### Models and statistical methods

In order to study differences in diversity and richness between rabbits grouped according to farm environment and management that they received, two alpha diversity indexes (Shannon and the observed number of OTUs) were computed from the OTU table rarified to 10,000 sequences per sample with “phyloseq” R package [52]. The statistical method chosen to assess alpha diversity differences between these groups of animals was an analysis of variance that included a factor resulting from the combination of four factors (the farm where the animal was raised, the batch, the presence or the absence of antibiotics in the feed and the feeding regime). The significance threshold was set at 0.05 for type I error.

Different approaches were considered to assess the influence of the environments and management factors on microbial composition. A bootstrap analysis of variance was individually implemented for each OTU to test whether it was differentially represented between the different categories of the factors studied. This univariate analysis was conducted by normalizing the OTU table with the cumulative sum scaling (CSS) method [53] and only for those OTUs which were detected in at least 5% of the samples and had a sum of its counts resulting in a frequency greater than 0.01% of the total sum of all OTUs counts across all samples. It was implemented by fitting a model defined by the combination of the four aforementioned factors by using *lm()* function in R [54]. Then, the differences between the CSS-normalized OTUs counts in the different levels of the studied factors were tested. The significance between the levels of the main factors: farm, presence of antibiotics in the feed and feeding regime was assessed using an F statistic. When the involved interaction terms were significant, the contrasts of interest were studied nested within the levels of other interacting factors, i.e. feeding regime were studied within farm levels. When the interaction terms were not significant, the effects of the different levels were averaged, i.e. the effects of the levels of the batches within farm A were averaged to present the effect associated with this farm. In the performed F tests, instead of relying on the theoretical distribution of the statistic under the null hypothesis to define the *p*-values, they were empirically computed using bootstrap after 1000 permutations of the dependent variable with respect to the design matrix of factors in the model. The use of bootstrapping enabled the hypothesis test to be done without the necessity of assuming that data are normally distributed, which is an assumption that fails for OTUs counts. *P*-value was defined as the proportion of bootstrap rounds having an F statistic value equal to or greater than that obtained with the original dataset. *P*-values were corrected defining a false discovery rate (FDR) of 0.05 [55]. This bootstrap analysis of variance approach was also implemented in order to study the effect of the management factors on the relative abundance of bacteria at phylum and genus levels.

The value of the microbial information to classify samples into the three factors considered in our study was explored using multivariate techniques. In particular, sparse partial least squares-discriminant analysis (sPLS-DA) [56] was used to find the combination of OTUs that allowed the best classification of cecal samples according to: (1) the farm where the animals were raised, (2) the feeding regime within farm A and (3) the combination of feeding regime and the presence or absence of antibiotics in the feed for the animals raised in farm B. This approach was implemented through the R package “mixOmics” [57]. In a first step, the function *tune.splsda()* was used to select the optimal sparsity parameters of the sPLS-DA model: the number of components and the number of variables (OTUs) per component. For the tuning process, a 5-fold cross-validation repeated 10 times was performed one component at a time, with a maximum of 4 components, on an input grid of values that indicate the number of variables to select on each component. The sparsity parameters were defined, based on the BER and centroids distance, and then included in the final sPLS-DA model. Samples were represented on the first two components and colored according to their class (e.g., R or AL in the case of the feeding regime) in a sample plot with the function *plotIndiv()*. The performance of the sPLS-DA model was assessed with a 5-fold cross-validation repeated 1000 times that randomly split the data in training and validation sets. In this data partition, it was ensured that 20% of the samples within each level of the discriminant factor were assigned to the validation set. Five different partitions were performed for each replicate to guarantee a different sample distribution in each validation set. The sPLS-DA model with the sparsity parameters previously defined was adjusted in the training set and its classification performance was assessed in the validation set using the overall and BER per class as criteria. The stability of the OTUs selected on each component was also assessed in the cross-validation by computing the selection frequency of each variable across the replicates.

## Supplementary Information

**Additional file 1.** Metadata associated with the 425 rabbit cecal samples analyzed in this study.

**Additional file 2.** Prefiltered and unnormalized OTU table used for statistical analyses in this study.

**Additional file 3.** Filtered and CSS-normalized OTU table used for statistical analyses in this study.

**Additional file 4.** Taxonomic assignments for all OTUs in Additional file 2.

**Additional file 5.** Relative abundances phyla table built from the collapse of the filtered and CSS-normalized OTU table at phylum level.

**Additional file 6.** Relative abundances genera table built from the collapse of the filtered and CSS-normalized OTU table at genus level.

**Additional file 7: Table S1.** OTUs differentially represented between farms.

**Additional file 8: Table S2.** OTUs differentially represented between feeding regimes within farms.

**Additional file 9: Table S3.** OTUs differentially represented between the presence and the absence of antibiotics in the feed within farm B.

## Data Availability

The raw sequence data were deposited in the sequence read archive of NCBI under the accession number SRP186982 (BioProject PRJNA524130). Metadata, the prefiltered and unnormalized OTU table, the filtered and CSS-normalized OTU table and corresponding taxonomic assignments have all been included as Additional files 1, 2, 3 and 4, respectively. Relative abundances phyla and genera table have also been included as Additional files 5 and 6, respectively. OTUs differentially represented between the studied factors, their sequences and their assignment at the lowest taxonomic level have been included as Additional files 7, 8 and 9.

## Abbreviations

AL: Ad libitum
BER: Balanced error rate
CSS: Cumulative sum scaling
FDR: False discovery rate
GIT: Gastrointestinal tract
NGS: Next generation sequencing
OTU: Operational taxonomic unit
PCR: Polymerase chain reaction
R: Restricted
sPLS-DA: Sparse partial least squares-discriminant analysis
SRB: Sulfate-reducing bacteria (SRB)
SSB: Sulfate-secreting bacteria (SRB)
